# How Azide Ion/Hydrazoic Acid Passes Through Biological Membranes: An Experimental and Computational Study

**DOI:** 10.1007/s10930-023-10127-3

**Published:** 2023-06-08

**Authors:** Simona Lojevec Hartl, Simon Žakelj, Marija Sollner Dolenc, Vladimir Smrkolj, Janez Mavri

**Affiliations:** 1 National Institute of Chemistry, Center for Validation Technologies and Analytics, Ljubljana, Slovenia; 2 University of Ljubljana, Faculty of Pharmacy, Ljubljana, Slovenia; 3 University of Ljubljana, Faculty of Medicine, Institute of Anatomy, Ljubljana, Slovenia; 4 National Institute of Chemistry, Laboratory of Computational Biochemistry and Drug Design, Ljubljana, Slovenia

**Keywords:** Azide ion, Hydrazoic acid, octanol/water partition coefficient, Effective permeability, PAMPA, Diffusion, Inhibition, Cytochrome c oxidase complex IV

## Abstract

Hydrazoic acid (HN_3_) and its deprotonated form azide ion (N_3_^−^) (AHA) are toxic because they inhibit the cytochrome c oxidase complex IV (CoX IV) embedded in the inner mitochondrial membrane that forms part of the enzyme complexes involved in cellular respiration. Critical to its toxicity is the inhibition of CoX IV in the central nervous system and cardiovascular system. Hydrazoic acid is an ionizable species and its affinity for membranes, and the associated permeabilities, depend on the pH values of aqueous media on both sides of the membranes. In this article, we address the permeability of AHA through the biological membrane. In order to understand the affinity of the membrane for the neutral and ionized form of azide, we measured the octanol/water partition coefficients at pH values of 2.0 and 8.0, which are 2.01 and 0.00034, respectively. Using a Parallel Artificial Membrane Permeability Assay (PAMPA) experiment, we measured the effective permeability through the membrane, which is logP_e_ − 4.97 and − 5.26 for pH values of 7.4 and pH 8.0, respectively. Experimental permeability was used to validate theoretical permeability, which was estimated by numerically solving a Smoluchowski equation for AHA diffusion through the membrane. We demonstrated that the rate of permeation through the cell membrane of 8.46·10^4^ s^−1^ is much higher than the rate of the chemical step of CoX IV inhibition by azide of 200 s^−1^. The results of this study show that transport through the membrane does not represent the rate-limiting step and therefore does not control the rate of CoX IV inhibition in the mitochondria. However, the observed dynamics of azide poisoning is controlled by circulatory transport that takes place on a time scale of minutes.

**Supplementary Information:**

The online version contains supplementary material available at 10.1007/s10930-023-10127-3.

## Introduction

Sodium azide (NaN_3_) is a white to colourless, crystalline solid that is highly water-soluble, odourless and tasteless [1]. It has a variety of uses: in agriculture, for the production of pesticides and herbicides, as well as for pest control; in biomedicine, as a chemical preservative in aqueous diagnostic reagents; and in hospitals and laboratories, as a chemical preservative in biological fluids. Moreover, it is used for military purposes for the production of detonators and other explosives [2, 3]. The most common use of sodium azide is as a propellant in air bags in vehicles. In this capacity, sodium azide is thermally unstable and it rapidly decomposes to nitrogen gas when it reaches a temperature of 300 °C, causing rapid expansion of the air bag [4]. In pharmacy, NaN_3_ is used as precursor in the synthesis of numerous compounds such as antiviral drugs, antibiotics [5], and a wide range of tetrazole derivatives, such as sartans. Sartan medicines (azilsartan medoxomil, azilsartan, candesartan cilexetil, candesartan, eprosartan, irbesartan, losartan, EXP3174, olmesartan medoxomil, olmesartan, telmisartan, valsartan [6]) are used as a long-term treatment for patients with arterial hypertension and those with certain heart or kidney diseases. Their use is well-documented in a recent review of the pharmacological treatment of arterial hypertension [7]. To a lesser extent, sartans are used concurrently in the treatment of diabetes, anticoagulant therapy, and cholesterol therapy [4]. Sartan medicines are known as antagonists of angiotensin II receptors and consequently block the action of angiotensin II, a hormone that constricts blood vessels and causes blood pressure to rise [8]. The active pharmaceutical ingredients of sartans and sartan medicines, contain residues of sodium azide in ppm levels as a result of the synthesis processes.

Although the genotoxic properties of sodium azide have been extensively studied, its in vivo genotoxic and mutagenic effects in mammalian cells are still controversial. The European Chemical Agency (ECHA) reports that sodium azide is a non-carcinogenic and non-mutagenic molecule [9] in mammalian cells based on a two-year rat study conducted in 1991. The U.S. Occupational Health and Safety Administration (OSHA) has classifed sodium azide as TLV-A4, “not classifiable as a human carcinogen” [10]. However, there are some studies that have demonstrated the genotoxicity or mutagenicity of sodium azide in mammalian cells. A study on *Drosophila melanogaster* larvae conducted in 1996 reports the induction of mitotic recombination in wing somatic cells of *Drosophila melanogaster* larvae after chronic exposure to sodium azide [11]. Raicu and Mixich reported in 1992 that sodium azide encapsulated in liposomes produced chromosomal aberrations when introduced into human heteroploid HFp-2 cells [12]. Jones et al. examining azide mutagenicity towards several rodent cell lines concluded that sodium azide is a mutagen for rodent cells [13]. The European Medicines Agency, a regulatory body, places the Threshold of Toxicological Concern (TTC) at 1.5 µg day^−1^ per person for intakes of genotoxic or mutagenic impurities, which is considered to be an acceptable risk level (excess cancer risk of < 1 in 100,000 over a lifetime of exposure) for most pharmaceuticals [14]. The United States Pharmacopoeia (USP) sets a concentration of NMT (not more than) 10 ppm of azide for sartans [15].

Chang et al. [16] pointed out that hypotension is the most common symptom of poisoning with sodium azide. Acute poisonings can occur with ingestion of sodium azide or with inhalation of vapours of hydrazoic acid (HN_3_) that are formed when sodium azide is dissolved in an acidic aqueous solution. At lower heart rates, tissues are less well-supplied with oxygen. Tissues that consume the largest amount of oxygen are muscles (20%), liver (20%), brain (20%), heart (10–12%), and kidney (7.2%) [17]. Damage to neurons is particularly critical since, in contrast to most other cell types, they do not easily regenerate and are not simply replaced after damage.

Sodium azide is primarily a mitochondrial toxin, as it binds within the electron transport chain (ETC), thus inhibiting oxidative phosphorylation [18]. The azide anion (N_3_^−^) has a greater affinity for the heme complex than O_2_ [19]. Therefore, it covalently binds to the fully oxidized form of iron (Fe^3+^), in place of O_2_, in the binuclear complex of *heme a*_*3*_ ${(\text{F}\text{e}}_{\text{h}\text{e}\text{m}\text{e} \text{a}3}^{3+}-{\text{C}\text{u}}_{\text{B}}^{2+})$ in the cytochrome c oxidase complex IV (CoX IV) bound to the inner mitochondrial membrane [20]. In CoX IV, the azide anion and trivalent iron form a bridging structure of ${\text{F}\text{e}}_{\text{h}\text{e}\text{m}\text{e}\,\text{a}3}^{3+}-\text{N}=\text{N}=\text{N}-{\text{C}\text{u}}_{\text{B}}^{2+}$ [21]. The resulting reduction in adenosine triphosphate (ATP) production, even in the presence of oxygen, results in metabolic failure (See Fig. S1, Fig. S2 and Fig. S3 under Supplementary Materials for a schematic representation of the normal ETC in Complex IV and a representation of the azide anion binding to the CoX IV complex in ETC [22, 23] )

When sodium azide dissolves in water or in an acidic medium it dissociates to sodium and azide ions. In acidic solution, the azide ion binds the proton and forms a neutral, non-ionizable and volatile hydrazoic acid (HN_3_). The chemical reaction takes place according to the following mechanism:

$$NaN_{3} + H_{2} O \to Na^{ + } + N_{3}^{ - } + H^{ + } + OH^{ - }$$

$${N}_{3}^{-}+{H}^{+}\to H{N}_{3}$$

HN_3_ is a weak acid with a pK_a_ value of 4.65 [18]. In acidic solution, neutral HN_3_ distributes preferentially to the octanol phase rather than to water. In basic solution, azide takes the deprotonated form of the azide anion, N_3_^− ^, which is distributed mainly in the aqueous phase when an octanol/water equilibrium is established. The contribution of ionic species to K_ow_ (octanol/water partition coefficient) is basically negligible in the case of HN_3_/N_3_^−^, while it becomes significant in the case of lipophilic species. For example, the local anesthetic bupivacaine, a lipophilic molecule, prefers the octanol environment over the acidic aqueous environment in its protonated form [24].

In this study, we addressed the permeability of AHA through the biological membrane. In order to understand the affinity of the membrane for the neutral and ionized form of azide we measured the octanol/water partition coefficients at pH values of 2.0 and 8.0, respectively. We measured the effective permeability through the membrane using a PAMPA experiment. By numerically solving the diffusion equation, we demonstrated that the measured permeability is at least to some extent dependent on diffusion through the unstirred water layer on each side of the membrane. Based on these experiments, we tried to determine whether diffusion through the membrane is the limiting step in the inhibition of the complex.

## Materials and methods

### Determination of the Octanol/Water Partition Coefficients (K_ow_) of Azide Ion/Hydrazoic Acid for Different pH Values Using Reversed-Phase Liquid Chromatography and UV Detection

In our study, we considered experimental octanol water partition coefficients for azidic acid and azide ion, respectively. In principle, one can apply one of the developed constant pH simulation algorithms [25, 26], but it should be noted that the HN_3_ pK_a_ value at the interface is highly dependent on the coordinate and is computationally as demanding as determining pK_a_ values in the protein interior [27, 28] and in contrast to proteins inaccessible to experiment [29].

Since hydrazoic acid is an ionizable species its octanol/water partition coefficient (K_ow_) depends on pH. We therefore decided to measure K_ow_ for two pH values and apply an extrapolation approach to determine the rest of the pH values [30]. The approach of Hodges et al. [31] is very similar to the approach of Strichartz et al. and both approaches consider contributions of both neutral and ionized species to the octanol/water partition coefficient. The determination of octanol/water (octanol/water) partition coefficients (K_ow_) of AHA was carried out using two different pH values of azide solutions (pH 2.0 and 8.0) and a reversed-phase liquid chromatography with UV detection. The pH-dependent partition coefficients K_ow_(pH) were calculated from the experimental data using the equation:

1 $$ K_{{ow}} (pH) = \frac{{[A]_{{oct}} }}{{[A]_{{aq}} }} $$

where [A]_oct_ represents total azide concentration (sum of neutral HN_3_ and deprotonated N_3_^−^ species) in the octanol organic phase and [A]_aq_ represents the total azide concentration in the aqueous phase.

Since the HPLC method does not distinguish between neutral and ionizable species of hydrazoic acid and azide anion, the partition coefficient K_ow_(pH) below can be expressed as a distribution coefficient Q(pH). Azide exists in its neutral or protonated form both in aqueous solution and in octanol. The ratio of concentrations of azide in octanol (the organic phase) and in water regardless of its protonation state at the given pH value of the environment is described by the distribution coefficient Q. Q(pH) is an equilibrium constant and a measure of free energy. We define Q(pH) as:

2 $$Q\left( {pH} \right) = \frac{{\left[ {A^{0} } \right]_{{org}} ~ + ~\left[ {A^{ - } } \right]_{{org}} ~}}{{\left[ {A^{0} } \right]_{{aq}} + ~\left[ {A^{ - } } \right]_{{aq}} }}$$

where [A^0^]_org_ stands for the concentration of the neutral form of azide (HN_3_, hydrazoic acid) in the organic phase (octanol), [A^0^]_aq_ for the concentration in the aqueous solution, [A^¯^]_org_ for the concentration of the deprotonated form of azide (N_3_^¯^) in the organic phase, and [A^¯^]_aq_ for the concentration of azide in the aqueous solution.

By expanding the numerator and denominator of the right-hand side of the Eq. (2) by factor 1/[A^−^]_aq_ one obtains

3 $$Q = \frac{{\left[ {A^{0} } \right]_{{org}} /\left[ {A^{ - } } \right]_{{aq}} + ~\left[ {A^{ - } } \right]_{{org}} /\left[ {A^{ - } } \right]_{{aq}} ~}}{{\left[ {A^{0} } \right]_{{aq}} /\left[ {A^{ - } } \right]_{{aq}} ~ + ~1}}$$

If a new variable P^0^ is introduced as the partition coefficient of a neutral species, then P^0^ = [A^0^]_org_/[A^0^]_aq_, and P^¯^ as the partition coefficient of deprotonated species is P^¯^ = [A^¯^]_org_/[A^¯^]_aq_, meaning the equation simplifies to a more compact form:

4 $$Q = \frac{{P^{0} \left[ {A^{0} } \right]_{{aq}} /\left[ {A^{ - } } \right]_{{aq}} ~ + ~P^{ - } ~}}{{~\left[ {A^{0} } \right]_{{aq}} /\left[ {A^{ - } } \right]_{{aq}} + ~1}}$$

[A^0^]_aq_/[A^−^]_aq_ is the equilibrium constant of neutral and deprotonated species in aqueous solution that depends both on the pK_a_ value of azide and the aqueous phase pH value.

The free energy for azide deprotonation (ΔG) with a given pK_a_ in a solution with a given pH value is:

5 $$ \Delta G = \ln 10 \cdot k_{b} T \cdot \left( {pH - pK_{a} } \right) $$

where *k*_*B*_ is Boltzmann’s constant and equals 1.987 kcal (mol K)^−1^ and T is the absolute temperature in Kelvins (310 K under physiological conditions). Equation 5 implies that an ionizable group with a certain pK_a_ value is in contact and in equilibrium with the aqueous solution at a certain pH value. There is a one-to-one correspondence between the equilibrium constant and free energy [32], which is expressed as

6 $$\Delta {\text{G}} = - {\text{k}}_{{\text{b}}} {\text{T}} \cdot \ln \left( {\frac{{\left[ {{\text{A}}^{0} } \right]_{{{\text{aq}}}} }}{{\left[ {{\text{A}}^{ - } } \right]_{{{\text{aq}}}} }}} \right)$$

By combining Eqs. 5 and 6 and by considering the equation ${\text{ln}}x = {\text{ln}}10 \cdot {\text{log}}x$, and introducing the relationship $\text{log}\beta =pH-{pK}_{a}$ or $\beta ={10}^{{\text{p}\text{K}}_{\text{a}}-\text{p}\text{H}}$, it is possible to write an equation for the azide distribution coefficient as a function of pH and pK_a_.

7 $${\text{Q}} = \frac{{{\text{P}}^{0} + \beta {\text{P}}^{ - } }}{{1 + \beta }} = \frac{{{\text{P}}^{0} 10^{{{\text{pK}}_{{\text{a}}} - {\text{pH}}}} + {\text{P}}^{ - } }}{{1 + 10^{{{\text{pK}}_{{\text{a}}} - {\text{pH}}}} }}$$

The distribution coefficient Q is the equilibrium constant for azide distribution between the organic phase and the aqueous phase regardless of the azide protonation state. The corresponding free energy ΔG associated with azide transfer from the aqueous solution with a certain pH value to the membrane is:

8 $$\Delta {\text{G}} = - {\text{k}}_{{\text{b}}} {\text{T}} \cdot \ln {\text{Q}} = - {\text{k}}_{{\text{b}}} {\text{T}} \cdot \ln \frac{{{\text{P}}^{0} + \beta {\text{P}}^{ - } }}{{1 + \beta }} = - {\text{k}}_{{\text{b}}} {\text{T}} \cdot \ln \left( {\frac{{{\text{P}}^{0} {\mkern 1mu} 10^{{{\text{pK}}_{{\text{a}}} - {\text{pH}}}} + {\text{P}}^{ - } }}{{1 + ~10^{{{\text{pK}}_{{\text{a}}} - {\text{pH}}}} }}} \right)$$

Since extracellular fluid (ECF) and cytoplasm (CYT) have different pH values of 7.4 and 7.2, respectively, the free energy for the transfer of AHA is not zero. Please note that both pH values refer to human cells [33, 34]. AHA tends to concentrate in the compartment with a more favourable solvation free energy. To calculate the relative population of AHA in the cell membrane, the appropriate Q for a given pH value is used:

9 $$\left[ A \right]_{{~memb\left( {pH_{1} } \right)}} = Q_{{\left( {pH_{1} } \right)}} \cdot \left[ A \right]_{{~ECF\left( {pH_{1} } \right)}} = Q_{{\left( {pH_{1} } \right)}}$$

The same equation can be used for the equilibrium between the cytoplasm and the membrane. By combining these two relative populations of AHA in the cytoplasm and the extracellular fluid (ECF), we get:

10 $$\left[ A \right]_{{~CYT\left( {pH_{2} } \right)}} = \frac{1}{{Q_{{\left( {pH_{2} } \right)}} }} \cdot \left[ A \right]_{{~memb\left( {pH_{1} } \right)}} = \frac{{Q_{{\left( {pH_{1} } \right)}} }}{{Q_{{\left( {pH_{2} } \right)}} }}$$

The relative AHA population in the cytoplasm is therefore simply the ratio of both Q.

By inserting Eq. 7, we can derive a simplified formula:

11 $$\left[ A \right]_{{~CYT\left( {pH_{2} } \right)}} = \frac{{Q_{{\left( {pH_{1} } \right)}} }}{{Q_{{\left( {pH_{2} } \right)}} ~}} = \frac{{10^{{pH_{1} - pK_{a} }} ~}}{{10^{{pH_{2} - pK_{a} }} }} = 10^{{pH_{1} - pH_{2} }}$$

As seen in Eq. 11, the overall AHA concentration in the cytoplasm is pK_a_ independent and depends only on the pH value difference of the cytoplasm and ECF.

### Determination of Effective Permeability, *P*_*e*_*(pH*) Using the PAMPA Method

**Fig. 1:**
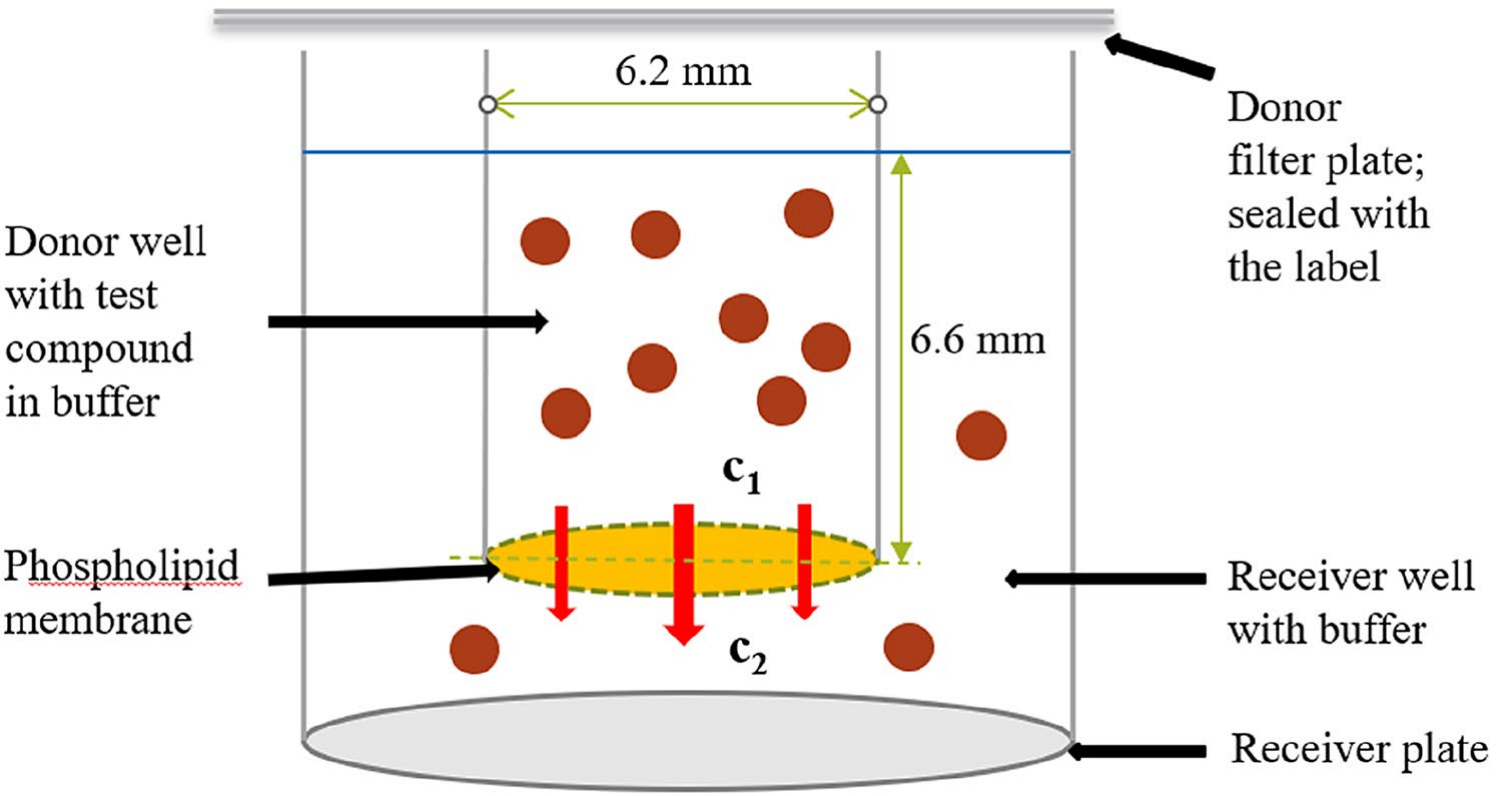
Schematic representation of a single well in the PAMPA sandwich. 200 µL of solvent per well was pipetted into upper donor plate and 300 µL of sample per well was pipetted into acceptor plate. The upper donor plate with sample was sealed with a label to prevent hydrazoic acid from evaporating and cross-contaminating other wells. We placed the filter plate on the receiver plate and incubated the assembly at room temperature for 5 h without stirring

Simulation of azide partition between the extracellular fluid and biological membrane was performed using the PAMPA (Parallel Artificial Membrane Permeability Assay) method, the schematics of which are shown in Fig. 1. PAMPA is an in vitro tool for high-throughput prediction of in vivo drug permeability and is also useful for the assessment of passive transport mechanisms [35]. Our experiments were performed using the Corning BioCoat Pre-Coated PAMPA Plate System with 96-well insert system with a 0.45 μm PVDF (polyvinylidene fluoride) filter plate which has been pre-coated with structured layers of phospholipids and a matched receiver microplate. The thickness *d* of the phospholipid membrane was 125 μm = 1,250,000 Å.

PAMPA experimental conditions are described in Table S3 under Supplementary Materials. Prior to use, the pre-coated PAMPA plate system was warmed to room temperature for 60 min. The compound solutions of NaN_3_ with concentrations of 0.241 and 0.120 mg mL^−1^ were prepared by dilution of stock solutions (1.2 mg mL^−1^) into a PBS buffer adjusted to two pH values of 7.4 and 8.0 using 1 M NaOH. 300 µL of solvent per well was pipetted into the acceptor plate and 200 µL of sample per well was pipetted into the upper donor plate. The upper donor plate with sample was sealed with the Sealing Tape for 96-Well Plates to prevent hydrazoic acid from evaporating and contaminating other wells. We placed the filter plate on the receiver plate and incubated the assembly at room temperature for 5 h without stirring. After that, we separated the plates and determined the compound concentrations in both plates using HPLC with UV detection; the chromatographic conditions are listed in Table S4 under Supplementary Materials.

Determination of effective permeability, *P*_*e*_*(pH*) for azide (cm s^−1^) at pH values of 7.4 and 8.0 was calculated as follows:

12 $$P_{e} = \frac{{ - ln\left[ {1 - C_{A} \left( t \right)/C_{{equilibrium}} } \right]}}{{S*\left( {\frac{1}{{V_{D} }} + \frac{1}{{V_{A} }}} \right)*t}}$$

We calculated Mass Retention (%) using Eq. 13:

13 $$1 - \frac{{\left[ {C_{D} \left( t \right) \cdot V_{D} + C_{A} \left( t \right) \cdot V_{A} } \right]}}{{C_{0} \cdot V_{0} }}$$

where *C*_*0*_ is the initial azide concentration in the donor well (mM), *C*_*D*_*(t)* is the azide concentration in the donor well at time *t* (mM), *C*_*A*_*(t)* is the azide concentration in acceptor well at time t (mM), *V*_*D*_ is the donor well volume of 0.2 mL (including compound in buffer), *V*_*A*_ is the acceptor well volume of 0.3 mL (including buffer), *S* is the filter area of 0.3 cm^2^, and *t* is the incubation time (5 h = 18,000 s). Equilibrium concentration, *C*_*equilibrium*_ (µM), was calculated as

14 $$C_{{equilibrium}} \frac{{\left[ {C_{D} \left( t \right) \cdot V_{D} + C_{A} \left( t \right) \cdot V_{A} } \right]}}{{V_{D} + V_{A} }}$$

### Theoretical Prediction of Permeability (P_t_)

Herman J.C. Berendsen was among the first to perform molecular simulations of membranes to address their structure, stability, and transport properties [36–41], followed by his disciples who included simulation of membrane embedded transporters [42–46].

Diffusion is a passive process of the net movement of a substance driven by a concentration gradient. Diffusion is described by the following diffusion equation in n-dimensions:

15 $$\begin{array}{c}\frac{\partial u}{\partial t}=D{\nabla }^{2}u\end{array}$$

where *u* is the coordinate and time-dependent concentration, *D* is the diffusion coefficient, and ${\nabla }^{2}$ is the Laplace operator. It must be noted that in our calculation *D* is coordinate- and time- independent. Furthermore, in complex environments, such as those in a nerve cell, the concentration difference is not the only force driving diffusion. Therefore, we include the coordinate-dependent chemical potential ($\mu$) in Eq. 15, which alters the time-dependent concentration profiles. This is a special case of the more general Fokker-Planck equation, called the Smoluchowski equation [47], which reads:

16 $$\frac{{\partial u}}{{\partial t}} = \nabla \left[ {D\left( {\nabla u + \frac{1}{{k_{b} T}}\left( {u \cdot \nabla \mu } \right)} \right)} \right]$$

where $\mu$ is the coordinate-dependent chemical potential, *k*_*b*_ is the Boltzmann constant, *T* is the absolute temperature, and $\nabla$ is the gradient of a function.

Since the diffusion equation with coordinate-dependent chemical potential described above does not allow for analytical solution, we proceeded to solve it numerically on a grid. Algorithms for numerical solutions of such a diffusion equation have appeared only recently [48]. The carefully performed computational study of oxygen diffusion through membranes by Ghysels et al. is particularly illustrative [49]. We have used the finite differences numerical method. Two numerical simulations were made, one with the dimensions of a realistic cell membrane with thickness of 50 Å and one with real PAMPA membrane thickness of 125 μm according to the manufacturer. The first experiment used grid spacing of 1 Å and a time step of 0.1 ns, while the second experiment used grid spacing of 1 μm and time step of 1 µs. In both simulations a diffusion coefficient D of 820 µm^2^ s^− 1^ from Huang and co-workers [50] was used. Moreover, both simulations used the free energy profiles of the transition into and out of the membrane, which were experimentally determined earlier in this study.

The permeability across the membrane is described by the following equation:

17 $$ {\text{j  =  }} - {\text{ P}}\Delta {\text{c}} $$

where *j* is the flux, *P* is the membrane permeability, and $\varDelta c$ is the difference in concentration on both sides of the membrane. Flux was calculated numerically from the diffusion equation with coordinate-dependent chemical potential, while the concentration difference was initially 1.

### Preparation of Solutions

Solvents for the sample solutions were prepared by mixing acetonitrile and water in ratio 1:10 (V/V). Solvents were adjusted to pH 2.0 with 20% H_3_PO_4_ and to pH 8.0 with 0.01 M NaOH before filling to the calibration mark.

First, the calibration curves were prepared at five concentration levels: these were from 0.12 to 1.5 mg mL^−1^ of NaN_3_ (corresponding to 0.08 to 1.0 mg mL^−1^ of HN_3_) for a pH value of 2.0, and from 0.0019 to 0.12 mg mL^−1^ of NaN_3_ (corresponding to 0.0013 to 0.08 mg mL^−1^ of HN_3_) for a pH value of 8.0.

Aqueous sample solutions of NaN_3_ with pH values of 2.0 and 8.0 were also prepared. The concentration of the sample solution with a pH value of 2.0 was 1.5 mg mL^−1^ of NaN_3_ (equal to 1.0 mg mL^−1^ of HN_3_) and the sample solution with a pH value of 8.0 was 12 mg mL^−1^ (equal to 8.0 mg mL^−1^ of HN_3_). Equal volumes, 15 mL of the solution of NaN_3_ and 15 mL of 1-octanol, were then pipetted into a 50 mL separatory funnel and mixed well every 10 min for one hour to equilibrate. Finally, the upper organic and lower aqueous phase were sampled. The aqueous phase with a pH value of 8.0 was diluted by a factor of 1250. All solutions for the calibration curves and final solutions of the organic and aqueous phases were injected into an HPLC system.

## Results

### Determination of octanol/water Partition Coefficients (K_ow_)

The octanol/water partition coefficient is defined as the ratio of equilibrium concentrations of a given species between octanol and water. For ionizable species such as azide, the ratio depends on the pH value of the aqueous solution.

The octanol/water partition coefficients (K_ow_) were calculated from the experimentally determined concentrations of AHA in both phases: the organic phase of octanol and the water phase of a 10% solution of acetonitrile. K_ow_ was 2.01 for the pH value of 2.0 and 0.00034 for pH value of 8.0. The R-squared of the calibration curves with solutions of sodium azide were 0.974 for a pH value of 2.00 and 1.0 for a pH value of 8.0. We used Eq. 7 to interpolate and extrapolate experimental data and to calculate K_ow_(pH) = Q(pH).

Experimental results show that in an acid environment (pH 2.0) in which azide mostly takes its neutral form (HN_3_), 67% of azide is in the octanol phase. In a basic environment with a pH above pK_a_ 4.65, the azide mostly exists in its ionic form N_3_^ˉ^, so a lower partition coefficient and concentration in octanol is expected and indeed the experimentally determined value is 0.00034 (pH of 8.0) of azide in octanol. The experimental values of the corresponding calculated free Gibbs energies of activation ΔG^‡^(pH) for the transition of AHA from the aqueous to the organic phase are − 0.413 kcal mol^−1^ (pH 2.0) and 3.29 kcal mol^−1^ (pH 8.0) and were calculated using the equation ΔG^‡^ = ∆G_ow_ = − k_B_T ∙ ln K_ow_. Extrapolating from the experimental results, the distribution coefficients, *Q*, and free energies, *ΔG*, for the transfer of AHA were calculated and are listed in Table 1.

**Table 1:** Partition coefficients, K_ow_(pH), and free energies, ΔG, for the transfer of AHA from water to octanol

pH	Q = K_ow_	logQ	ΔG [kcal mol^− 1^]
1	2.00955	0.303	− 0.414
2	2.00551	0.302	− 0.413
3	1.96599	0.294	− 0.401
4	1.64239	0.215	− 0.294
4.65	1.00517	0.002	− 0.003
6	0.08627	− 1.064	1.453
6.8	0.01447	− 1.840	2.512
7.1	0.00745	− 2.128	2.906
7.2	0.00599	− 2.223	3.035
7.4	0.00391	− 2.408	3.288
8	0.00124	− 2.908	3.970
9	0.00043	− 3.367	4.597
10	0.00035	− 3.457	4.721

**Fig. 2:**
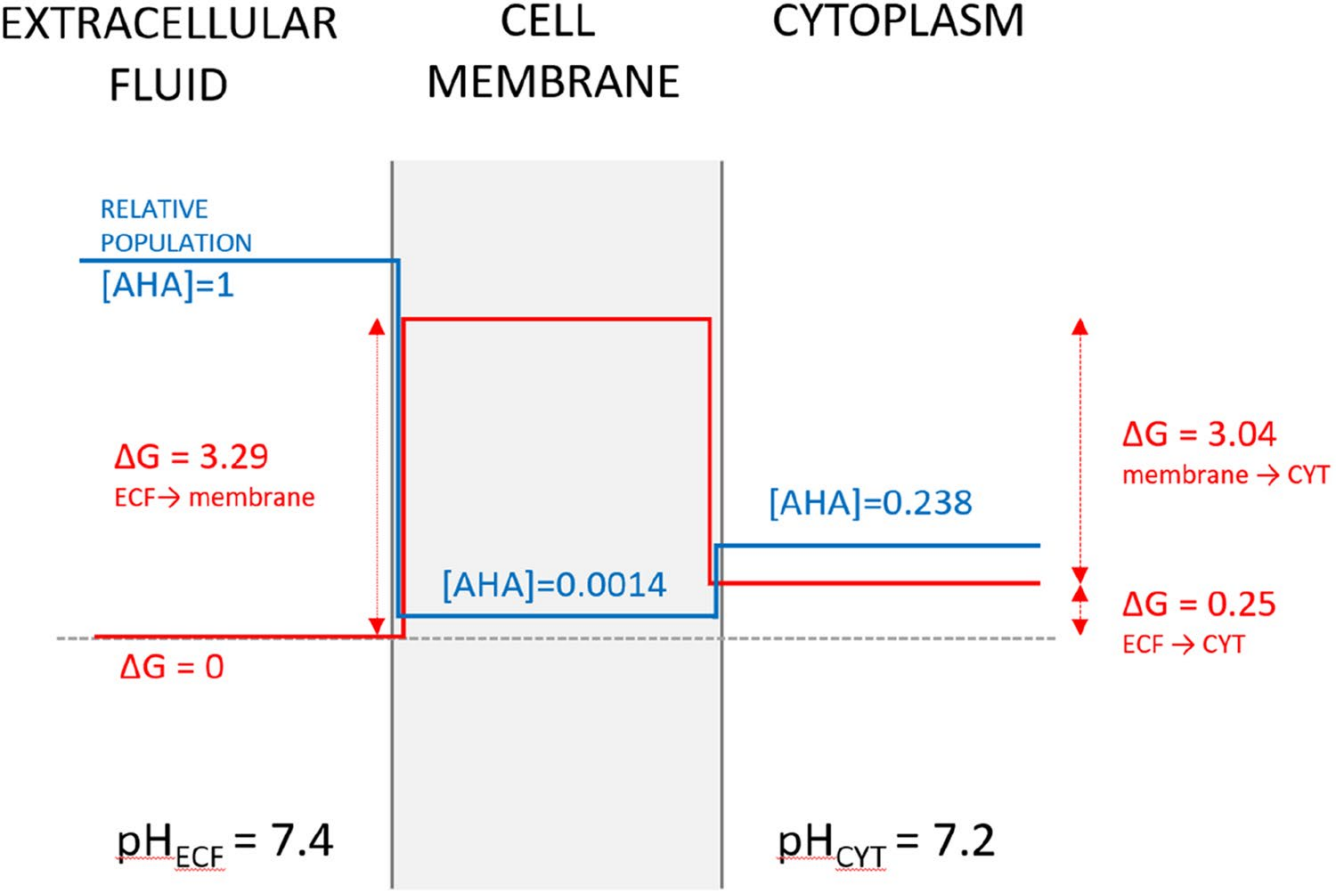
Free energy profile for transfer of AHA from extracellular fluid (ECF) to cytoplasm (CYT). Please note that the free energy difference between ECF and CYT is due to the different pH values on both sides of the membrane. Relative concentrations of AHA in the ECF, cell membrane and CYT are represented by the blue line. ECF is an endless reservoir with the relative concentration of AHA set to 1 and a pH_ECF_ value of 7.4. The pH_CYT_ value of the cytoplasm is 7.2. The red line represents the free energy profile for transfer of AHA from the ECF to CYT under physiological conditions. Total ΔG is the change in free energy between the two compartments (0.25 kcal mol^−1^). All values are given in kcal mol^−1^. Note that the free energy profile was constructed solely from the experimental data

Please refer to Fig. 2 to aid with understanding of the local environment around the cell membrane.

### Experimental Determination of Effective Permeability, P_e_ (pH)

The results of the effective permeability of the artificial membrane for AHA were obtained using the following approaches: experimentally, by using the PAMPA method, and, theoretically, by numerically solving the diffusion equation.

Experimentally, we obtained a value of P_e_ of 11.2 · 10^−6^ cm s^−1^ at a pH value of 7.4 and 5.5 · 10^−6^ cm s^−1^ at a pH value of 8.0 (see Table 2). Nearly 11% of azide crossed the membrane into the acceptor solution with a pH value of 7.4 and 9% into the acceptor solution with pH value of 8.0. Mass retention, calculated on the donor and acceptor side was on average − 0.15% and − 0.03% for pH 7.4 and 8.0, respectively, which indicates a minor analytical error.

**Table 2:** Experimental results of effective permeability (P_e_) for AHA for two pH values using the PAMPA method

Input data				Calculation results			
pH	Acceptor concentration (µM)	Donor concentration (µM)	Initial concentration (µM)	Equilibrium concentration (µM)	Mass retention (%)	Permeability P_e_ (cm s^− 1^)	Permeability logP_e_
7.4	675.5	3247.7	3700.9	1704.4	− 0.15	11.2 · 10^− 6^	− 4.97
8.0	329.9	3296.8	3696.3	1516.6	− 0.03	5.5 · 10^− 6^	− 5.26

### Computational Prediction of Permeability (P_t_)

By solving the diffusion equation numerically, we obtained two permeability values P_t_ (logP_t_) for the AHA transition from the ECF to CYT (Fig. 2) of 4.23 ·10^−2^ cm s^−1^ (− 1.73) and 22.3 ·10^−6^ cm s^−1^ (− 4.65), for 50 Å and 125 μm membrane, respectively.

Experimental (− 4.97) and theoretical values (− 4.65) of permeability at a physiological pH of 7.4 for the 125 μm PAMPA membrane differ only slightly. However, the realistic 50 Å membrane has a much higher permeability (10^4^ times). Similarly, we can calculate the theoretical rate constant of permeation through the membrane, which is 8.46 · 10^4^ s^−1^ and 1.55 · 10^−3^ s^−1^ for the 50 Å and 125 μm membrane, respectively.

## Discussion

Current opinion is that azide enters the cell through the plasma membrane rather than via any of the transporters. In this respect it is very likely that it enters in its neutral form of HN_3_, since its deprotonated form (N_3_^−^) has a too-favourable free energy of hydration, consequently the free energy cost for entering the membrane would be too high. The free energy for protonation of ionized species with a given pK_a_ value in aqueous medium with a given pH value is $\varDelta G=-{ln}\left(10\right){k}_{B}T\left(pH-p{K}_{a}\right)$, *w*here $\varDelta G$ is the free energy, ${k}_{B}$ is the Boltzmann constant, *T* is the absolute temperature (310 K for human body) and the pK_a_ value for hydrazoic acid is 4.65. Hence, the free energy for azide protonation at pH of 7.4 is $- 2.303 \cdot 0.593\,{\text{kcal mol}}^{{ - 1}} \cdot (7.4 - 4.65) = - 3.75\,{\text{kcal mol}}^{{ - 1}}$. On the other hand, the energy cost for HN_3_ deprotonation in media with a pH value of 2.0 is 3.62 kcal mol^− 1^. Since deprotonated ionic species (N_3_^−^) have a more favourable hydration energy in more basic media, equilibrium populations of HN_3_ are higher in the cytoplasm (CYT, with a pH value of 7.2) than in the extracellular fluid (ECF, with a pH value of 7.4) or in the mitochondria (with a pH value of 8) and equilibrium populations of N_3_^ˉ^ are highest in the mitochondria.

At lower pH, azide mostly exists in the protonated form of hydrazoic acid (HN_3_), which is hydrophobic to some extent and preferentially distributes to the gas phase or lipid membrane from aqueous solution. Hydrazoic acid has a relatively small dipole moment of 0.847 ± 0.005 Debye [51]. Observing the measured octanol/water partition coefficient supports this. At pH 2, there is more AHA in the nonpolar solvent (2:1), while at pH 8 there is significantly more AHA in the water phase (1:2940). Consequently, within the physiological pH range, azide is always hydrophilic and predominantly in ionized form (N_3_^−^). According to the Overton rule [52] more hydrophilic substances are less membrane permeable [53, 54]. Consequently, one could predict that AHA will not permeate easily through the cell membrane. However, its small molecular size and the spread charge in its ionized form yield a moderately low free energy barrier of 3.29 kcal mol^−1^ for entry into the membrane. By comparison, MMB4, a popular drug that crosses the blood-brain barrier, has a free energy barrier of 28 kcal mol^−1^ [55], morphine has a free energy barrier of 7 kcal mol^−1^ [56], and carbon dioxide has a free energy barrier of around 1 kcal mol^−1^ [57]. AHA diffuses from the region with a higher pH value (ECF) to the region with a lower pH value. More azide molecules are in protonated form (HN_3_) in cytoplasm with a pH value of 7.2, than in the ECF at a higher pH value of 7.4, consequently the equilibrium free energy difference for permeation from the ECF to the cytoplasm is 0.25 kcal mol^−1^, which is solely due to the difference in pH. Local acidosis or alkalosis would change this. More interesting is that the equilibrium concentration ratio between the cytoplasm and the ECF changes depending on the pH difference. In the case of physiological pH values, less AHA is in cells than in the ECF (0.238), while in the case of local acidosis, additional AHA would accumulate in cells. For example, poisoning with azide disturbs the aerobic metabolism of the cell, shifting it to anaerobic, which produces acidic lactic acid, thus lowering the ECF pH and starting a positive feedback loop or death spiral.

Our experimental data for the permeability of AHA using the PAMPA method gave a permeability value (logP_e_) of − 4.97, which is still considered fast, according to Bennion et al., who estimate that compounds with logP_e_ < − 5.5 have low permeability [55]. Bodor et al. studied the correlation between the octanol/water partition ratio (Q) and blood-brain barrier permeability [58]. AHA with a Q of 0.00391 would have a low blood-brain barrier permeability similar to urea. It should be noted that PAMPA is specifically designed to study intestinal absorption of drugs, but we study permeation through the cell membrane, which is about 25,000 times thinner. Therefore, we conducted two simulations. The results of numerical solutions for the diffusion equation predict the experimental permeability for a membrane of 125 μm reasonably well. In fact, a surprisingly good agreement between the experimental and calculated values was found. However, currently there are no reliable experimental methods that would yield the permeability of a cell membrane with a thickness of approximately 50 Å. Since the computational predictions proved to be valid for a 125 μm thick membrane we firmly believe that the permeability predictions for a 50 Å membrane are equal reliable.

Given that the rate constant of permeation through the cell membrane for AHA is 8.46 ·10^4^ s^−1^ and the rate constant for the association of AHA with the Cytochrome c oxidase (CoX IV) is 200 s^−1^ as measured by Antalis et al. [59] for association with myoglobin, we have good evidence that the diffusion through the membrane is not the rate limiting step and does not control the rate of CoX IV inhibition by AHA. The chemical step, which is the reaction of the heme group when AHA forms a Michaelis complex with CoX IV, controls the overall rate of inhibition. The myoglobin association rate is similar to CoX IV because both have heme groups, which are targeted by the azide ion. The rate limiting step is therefore the association with heme, since AHA is a small molecule that can easily permeate the membrane regardless of its hydrophilic nature. It is worth stressing that for most enzymatic reactions in aqueous solution diffusion is rarely the rate-limiting step. The “average enzyme” is far from kinetic perfection as it exhibits a k_cat_ of ∼10 s^−1^ [60]. We are aware that the observed dynamics of azide poisoning is however controlled by circulatory transport that takes place on a time scale of minutes and to some extent depends on the route of administration (intravenous (IV), inhalation, intramuscular (IM)).

## Conclusions

By measuring the octanol/water partition coefficients for the azide ion/hydrazoic acid (AHA) we have demonstrated that, at physiological pH values of extracellular fluid, AHA prefers extracellular fluid over cytoplasm, while membrane and other lipophilic compartments have the lowest relative populations.

The experimental values for effective permeability obtained using the PAMPA method were compared with the results of numerical solution of the diffusion equation through the membrane. For a 50 Å thick membrane we estimated the permeability of AHA to be 8.46 · 10^4^ s^−1^ while the rate constant for the association of AHA with the heme group is 200 s^−1^ as measured by Antalis et al. [59]. One can therefore conclude that CoX IV inhibition by azide ion is controlled by the chemical step rather than by diffusion through the membrane. It should be emphasized that this study focused on the events that follow complete embedding of the cell in the azide ion/hydrazoic acid solution. However, the observed dynamics of azide poisoning are controlled by transport in the circulation, which takes place on a time scale of minutes.

## Supplementary Information

Below is the link to the electronic supplementary material.

Supplementary material 1 (PDF 650.7 kb)

## Data Availability

All data and code can be obtained from the authors upon request.
