# Supplementary material for: How Azide Ion/Hydrazoic Acid Passes Through Biological Membranes: An Experimental and Computational Study
Source: Protein J. 2023 Jun 8;42(3):229–38. doi: 10.1007/s10930-023-10127-3 (PMC10264542; doi:10.1007/s10930-023-10127-3)
Supplement: Supplementary file 1 — Supplementary material 1 (PDF 650.7 kb) [file 10930_2023_10127_MOESM1_ESM.pdf]

Supplementary information for

How Azide ion / Hydrazoic Acid Passes Through Biological Membranes: An Experimental and Computational Study

Simona Lojevec Hartl[1,2], Simon Žakelj[2], Marija Sollner Dolenc[2], Vladimir Smrkolj[3,*], Janez Mavri[4,*]

[1]*Center for Validation Technologies and Analytics, National Institute of Chemistry, Ljubljana, Slovenia*

[2] *University of Ljubljana, Faculty of Pharmacy, Slovenia*

[3] *University of Ljubljana, Institute of Anatomy, Faculty of Medicine, Slovenia*

[4]*Laboratory of Computational Biochemistry and Drug Design, National Institute of Chemistry, Ljubljana, Slovenia*

**Corresponding authors: Vladimir Smrkolj, Institute of Anatomy, Faculty of Medicine, University of Ljubljana, Slovenia, E-mail: vladimir.smrkolj@gmail.com*

Janez Mavri, National Institute of Chemistry, Slovenia, E-mail: janez.mavri@ki.si

1. Chemicals and reagents

All solutions were prepared using Milli Q water from a Millipore water purification system (electrical resistivity > 18.2 MOhm x cm). For mobile phase, calibration curve and sample solution, the following chemicals and reagents were used: ortho-phosphoric acid (85 %; Supelco, Switzerland), acetonitrile (for HPLC LC-MS grade; VWR, USA), sodium hydroxide (pellets, p.a.; Merck, Germany), sodium azide (≥ 99.5 %; Sigma-Aldrich, China), 1-octanol (≥ 99 %, anhydrous, Sigma-Aldrich, Germany) and chemicals for preparation of physiological buffer solution (PBS): potasium phosphate monobasic (p.a., Merck), potasium phosphate dibasic (p.a., Fluka) and sodium chloride (p.a., Merck). Before completion to the volume mark, solutions from Experiment 1 were adjusted to pH of 2.0 and 4.65 with 0.2 % H_3PO_4 and to pH 8.0 with 0.01 M NaOH. Solutions of PBS from Experiment 2 were adjusted to pH of 7.4 and 8.0 with 1 M NaOH.

2. Equipment

A 1290 Infinity UHPLC system from Agilent, Germany was used. The system consists of 1200 bar pump, Diode Array Detector (DAD) with a standard cell with ultra-sensitive cell with 60 mm optical path length, Autosampler, Thermostat and Thermostated Column Compartment TCC. The chromatographic data acquisition was done with OpenLab CDS EzChrom Edition.

Separations were performed using a Phenomenex Synergi Hydro RP analytical column, USA (250 x 4.6 mm, 4 µm). Sample solutions and solutions for calibration curves were prepared using an analytical balance model XPE205DR/M from Mettler Toledo, Switzerland and a pH meter Seven Excelence™, model S400 from Mettler Toledo, China.

Experiment 1: Determination of octanol-water partition coefficients (K_{ow}) of AHA for pH values of 2.00 and 8.00 were measured using reversed-phase liquid chromatography and UV detection. Instrumental chromatographic conditions are described in Tables S1 and S2.

3. Figures

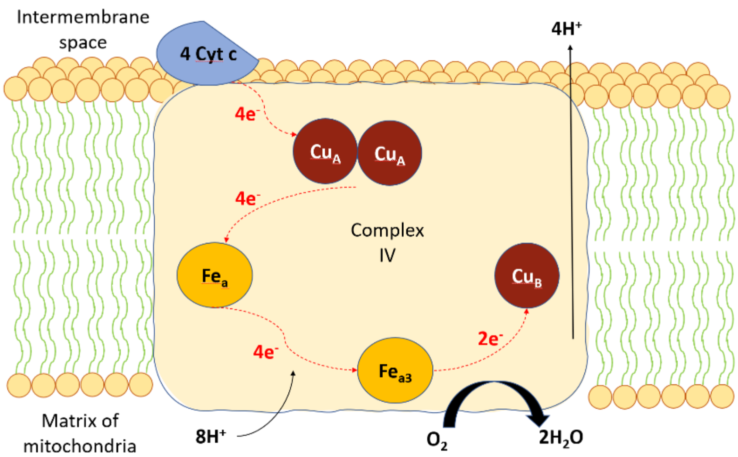

Fig. S1 Schematic presentation of usual process of electron transfer in Complex IV, the terminal enzyme in ETC of mitochondria. CoX IV contains two heme groups (*heme a* and *heme a3*; with Fe_a and Fe_{a3} in their centers) and three copper atoms (Cu_A/Cu_A and Cu_B). *Cytochrome c* (*Cyt c*) is a hemeprotein also and a carrier of electrons between Complex III and Complex IV. Complex IV reduces an oxygen molecule to 2 water molecules. 4 protons are delivered to the intermembrane space where they contribute to production of high energy molecule ATP. Whole reaction follows as:

$$4\,(\text{Cytochrome c})^{2+}_{\text{red}} + 8\,\text{H}^{+}_{\text{matrix}} + \text{O}_2 \rightarrow 4\,(\text{Cytochrome c})^{3+}_{\text{ox}} + 2\,\text{H}_2\text{O} + 4\,\text{H}^{+}_{\text{interm. space}}$$

This process returns the elements of complex CoX IV to their original states to begin another cycle.

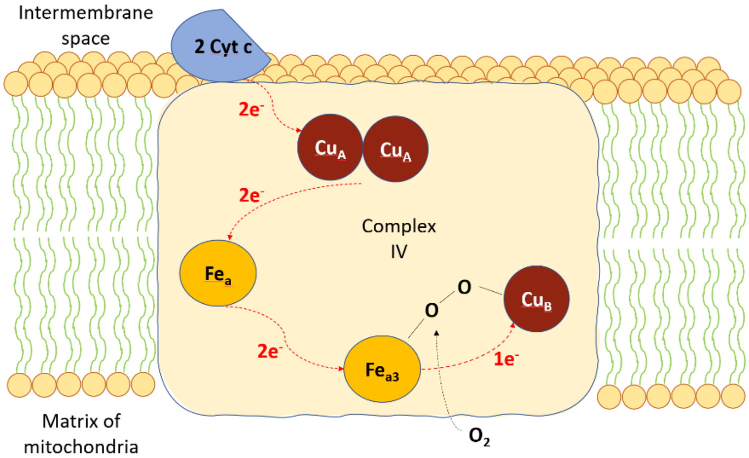

Fig. S2 Two *Cyt c* (attachments) bring 2 electrons, one for Fe_{a3} and one for Cu_B. The reduced Fe_{a3} and Cu_B allow an O_2 to bind and form a peroxide bridge. In further reaction two additional *Cyt c* attachments bring 2 more electrons and collecting $2H^+$ protons form hydroxides (Fe_{a3}-OH and Cu_B-OH). With another $2H^+$ protons the Fe_{a3} and Cu_B are reduced to their original states and as final state two water molecules are produced (see Fig. S1).

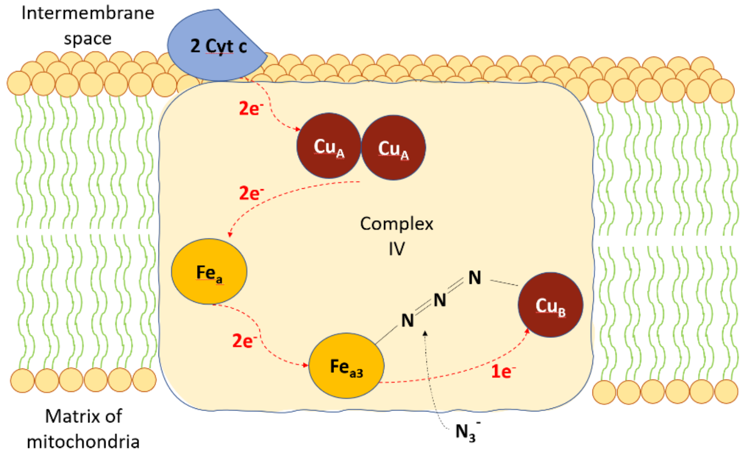

Fig. S3 Schematic presentation of azide anion binding to complex CoX IV in ETC. In presence of azide anion (N_3^-), peroxide bridge does not form due to higher affinity of N_3^- towards O_2. Instead, bridging structure of $Fe^{3+}_{\text{heme a3}} - N = N = N - Cu^{2+}_B$ is formed. If mitochondria cannot utilise O_2 as a final electron acceptor, complex CoX IV is inhibited and therefore, ATP is not formed. Lack of ATP follows to metabolic failure and cell death.

4. Tables

Table S1 Chromatographic conditions for HPLC-UV applied to the azide assay in the aqueous and organic phase.

Column	Synergy Hydro RP analytical column (250 x 4.6 mm, 4 μm)
Mobile phase A	0.5 g L^{-1} H_3PO_4
Flow rate	1.5 mL min^{-1}
Column temperature	30 °C
Detection	205 nm
Injection volume	20 μL
Run time	17 min
Solvent of sample solution	Acetonitrile: water = 1:10 (V/V), adjusted to pH 2.00, 4.65 and 8.00

Table S2 Gradient conditions

t (min)	Mobile phase A (%)	Mobile phase B (%)
0	100	0
4.5	100	0
5	0	100
10	0	100
10.5	100	0
17	100	0

Determination of effective permeability, Pe (pH)

Determination of effective permeability, P_e *(pH)* was carried out using method PAMPA at pH values of 7.4 and 8.0; we tried to determinate P_e *(pH)* at acidic pH values (pH 2.0, 3.75) also, but experiments were not successful due to high volatility of HN_3 at low pH values and consequent cross-contamination with the other wells. Experimental conditions for PAMPA are described in Table S3. Determination of compound concentrations in both plates were carried out using RP-LC with UV detection; chromatographic conditions are listed in Table S4.

Table S3 PAMPA experimental conditions

Sample<br>Solvent<br>Volume per well, upper donor plate (filter plate) with sample	NaN_3; 0.241 and 0.120 mg mL^{-1}<br>PBS, adjusted to pH 7.4, 8.0<br>200 µL
Volume per well, acceptor plate with solvent	300 µL
Incubation time	5 h
Membrane thickness, d<br>Well diameter, 2r<br>Donor well height, h	125 µm = 1 250 000 Å<br>6.2 mm<br>6.6 mm
Determination of compound concentrations	HPLC-UV

Table S4 Chromatographic conditions of Experiment 2 for HPLC-UV applied to the azide assay in the donor and acceptor plate of PAMPA using isocratic method.

Column	Synergy Hydro RP analytical column<br>(250 x 4.6 mm, 4 µm)
Mobile phase A<br>Flow rate<br>Column temperature<br>Detection<br>Injection volume<br>Run time	0.5 g L^{-1} H_3PO_4<br>1.5 mL min^{-1}<br>30 °C<br>205 nm<br>20 µL<br>7 min
